# Pro-resolving and pro-inflammatory fatty acid-derived mediators in sputum of stable state bronchiectasis patients

**DOI:** 10.1186/s12931-022-02301-5

**Published:** 2022-12-20

**Authors:** Leonardo Terranova, Patrizia Risé, Andrea Gramegna, Christian Pinna, Carlo Agostoni, Marie-Louise Syrén, Stefano Turolo, Paola Marchisio, Francesco Amati, Stefano Aliberti, Angelo Sala, Francesco Blasi

**Affiliations:** 1 Internal Medicine Department, Respiratory Unit and Adult Cystic Fibrosis Center, Fondazione IRCCS Ca’ Granda Ospedale Maggiore Policlinico, Via Francesco Sforza 35, 20122 Milan, Italy; 2 Department of Pharmaceutical Sciences-DISFARM, University of Milan, 20122 Milan, Italy; 3 Department of Pathophysiology and Transplantation, University of Milan, 20122 Milan, Italy; 4 Pediatric Unit, Fondazione IRCCS Ca’ Granda Ospedale Maggiore Policlinico, 20122 Milan, Italy; 5 Department of Clinical Sciences and Community Health, University of Milan, 20122 Milan, Italy; 6 Pediatric Nephrology, Dialysis and Transplant Unit, Fondazione IRCCS Ca’ Granda Ospedale Maggiore Policlinico, 20122 Milan, Italy; 7 Pediatric Highly Intensive Care Unit, Fondazione IRCCS Ca’ Granda Ospedale Maggiore Policlinico, 20122 Milan, Italy; 8 Department of Biomedical Sciences, Humanitas University, 20072 Pieve Emanuele, Italy; 9 Respiratory Unit, IRCCS Humanitas Research Hospital, 20089 Rozzano, Italy

**Keywords:** Bronchiectasis, Docosahexaenoic acid, Arachidonic acid

## Abstract

**Background:**

Bronchiectasis is characterized by neutrophilic inflammation and frequent exacerbations often associated with infections. Lipid mediators play critical roles in the inflammatory response, and the balance between anti-inflammatory and pro-inflammatory mediators could drive to chronic inflammation. The aim of this study was to evaluate the metabolites of docosahexaenoic acid and arachidonic acid in sputum of adults with bronchiectasis defining their associations with clinical data, bacterial load and neutrophil elastase.

**Methods:**

An observational, cross-sectional study was conducted at the bronchiectasis program of the Policlinico Hospital in Milan, Italy, where patients were enrolled. Active neutrophil elastase was measured by enzyme-linked immunosorbent assay, pro-resolving and pro-inflammatory fatty acid-derived mediators were evaluated by mass spectrometry and respiratory pathogens were assessed by real-time PCR. Analysis were performed on sputum collected during stable state and clinical data were also collected.

**Results:**

Levels of pro-inflammatory mediators derived from arachidonic acid metabolism showed association with neutrophil elastase, were proportional to *Pseudomonas aeruginosa* identifications and were linked with radiological gravity index, while the concentrations of pro-resolution mediators derived from docosahexaenoic acid were associated with a better health status, highlighted by the inverse correlation with radiological gravity index, bacterial infections and sputum volume production.

**Conclusion:**

Pro-inflammatory mediators derived from FA metabolisms are associated with severity of bronchiectasis while DHA-derived metabolites are inversely associated with severity of the disease, which may be used for personized treatment of bronchiectasis.

## Introduction

Bronchiectasis is a chronic and debilitating respiratory disease characterized by lung inflammation and permanent bronchi dilatation. This condition affects peoples of all ages profoundly affecting the quality of life: subjects with bronchiectasis have daily cough, excessive sputum production and frequent pulmonary exacerbations [1], often associated with lung bacterial infections [2, 3]. Secretion of pro-inflammatory mediators from epithelial and immune cells typically causes a massive neutrophil influx within the airways, as often observed in frequent exacerbators colonized by *Pseudomonas aeruginosa (Psa)* [4]. Early neutrophil involvement with release of reactive oxygen species (ROS), pro-inflammatory lipid mediators (LMs) and protease such as neutrophil elastase (NE) is directly involved in lung damage in bronchiectasis [5]. Studies on cystic fibrosis (CF) have reported the presence of fatty acid alteration in the blood and tissues of patients, such as decreased levels of docosahexaenoic acid (DHA), and of linoleic acid (LA) [6] together with increased levels of arachidonic acid (AA). Fatty acids metabolites play multiple roles in the inflammatory response: AA metabolites possess both potent pro-inflammatory and anti-inflammatory activities, while DHA-derived molecules, such as maresins, protectins and resolvins, are involved in the resolution of inflammation and are collectively defined as specialized pro-resolving mediators (SPMs) [7–10]. We hypothesized that changes in the relative availability of polyunsaturated fatty acids (PUFAs) may lead to an altered balance of pro-inflammatory and pro-resolution lipid mediators, either resulting in, or as a result of chronic colonization by different types of bacteria, leading to chronic inflammation.

Based on this assumption the aim of this study was to evaluate the presence of metabolites derived from DHA and AA into the airways of adults with bronchiectasis during stable state, analyzing potential correlation with clinical and radiological parameters, neutrophil elastase activity and bacterial colonization.

## Material and methods

### Study design and population

An observational cross-sectional study was carried out at the Bronchiectasis Program of the Respiratory Department, Fondazione IRCCS Ca’ Granda Ospedale Maggiore Policlinico, Milan, Italy, between March 2017 and March 2019. Adults (aged ≥ 18 years) with clinically (daily sputum production) and radiologically (at least one lobe involvement on chest computed tomography) bronchiectasis were recruited during clinical stability (⩾1 month from the last exacerbation and antibiotic course). Patients with cystic fibrosis or bronchiectasis due to pulmonary fibrosis were excluded. The study was performed in accordance with the declaration of Helsinki, was approved by the ethical committee of the hospital (Ethics committee Milano Area 2, Fondazione IRCCS Ca’ Granda Ospedale Maggiore Policlinico, 255_2020) and all subjects provided written informed consent to participate.

### Study procedures

Spontaneous sputum samples were obtained, and mucus plugs were isolated. Deoxyribonucleic acid (DNA) was extracted according to a published technique [11, 12]. Aliquots of mucus plug were also diluted 8 × in PBS, vortexed and centrifuged at 4 °C for 15 min at 3000* g*. Supernatants and DNA were stored at − 80 °C until analysis.

### Mass spectrometry analysis of AA and DHA metabolites

After thawing of sputum supernatant samples (0.2-1 mL), aliquots (0.2 mL) were added with stable isotope labeled internal standards (leukotriene B_4_ [d_4_]LTB_4_; prostaglandin E_2_ [d_4_]PGE_2_; 5-hydroxyeicosatetraenoic acid [d_8_]5-HETE, and lipoxin A_4_ [d_5_]LXA_4_; 2.5 ng each), centrifuged, acidified with acetic acid (final concentration 0.01%) and extracted using preconditioned polymeric solid phase extraction cartridges (Strata-X, 33 µm Polymeric Reversed Phase; Phenomenex, Torrance, CA). After washing with ultrapure water, DHA- and AA-derived metabolites were eluted using methanol/water, 90/10, v/v (0.5 ml), and the eluate taken to dryness using a rotary vacuum evaporator (SpeedVac; Thermo Scientific, Waltham, MA). Upon reconstitution in 40 µL HPLC solvent A (8.3 mM acetic acid buffer to pH 5.7 with ammonium hydroxide) plus 20 µL of HPLC solvent B (acetonitrile/methanol, 65:35, v/v), an aliquot of each sample (20 µL) was injected onto a C18 HPLC column (Ascentis 150 × 2 mm, 3 µm; Supelco, Bellefonte, PA) and eluted at the rate of 400 µL/min with a linear gradient from 45% solvent B, which was increased to 75% in 12 min, to 98% in 2 min, then held for 11 min before re-equilibration at 45% B for 10 min. The HPLC effluent was directly infused into a triple quadrupole mass spectrometer (API4000, Applied Biosystem, Foster City, CA, U.S.A.) equipped with electrospray ion source for mass spectrometric analysis in the negative ion mode using multiple reaction monitoring for the specific *m/z* transitions: 343–281 for 17-hydroxy-docosahexaenoic acid (17OH-DHA, the precursor of both resolvins and protectin), 359–206 for protectin D1(PD1), 375–141 for resolvin D1 (RvD1), 343–205 for 14-hydroxy-docosahexaenoic acid (14OH-DHA, the precursor of maresin), 359–250 for maresin 1 (MaR1), 335–195 for leukotriene B_4_ (LTB_4_), 319–219 for 15-hydroxy-eicosatetraenoic acid (15-HETE), 351–271 for prostaglandin E_2_ (PGE_2_), 438–333 for leukotriene E_4_ (LTE_4_), 327–116 for [d_8_]5-HETE, 339–197 for [d_4_]LTB_4_, 359–275 for [d_4_]PGE_2_, 443–338 for [d_5_]LTE_4_, and 356–222 for [d^5^]LXA_4_, that was used as IS for resolvin D1 RvD1. Quantification was performed using area ratios to the corresponding internal standards, and data were analyzed using MassHunter software. Standard curves were obtained using synthetic PD1 (a gift from Dr. Thierry Durand, CNRS, Montpellier, France), LTB_4_, PGE_2_, RvD1, MaR1, 15-HETE, LTE_4_, 14OH-DHA and 17OH-DHA (Cayman Chem, Ann Arbor, MI). The peak–area ratios of every compound to the relevant deuterated internal standard was calculated and plotted against the amount of the synthetic standards. Calibration lines were calculated by the least squares linear regression method and the correlation coefficient r^2^ was always better than 0.99. To calculate the concentration of any given analyte, the peak–area ratio to the relevant internal standard was calculated and read off the corresponding calibration line. Detection limit varied between 1 and 25 pg injected (3 to 75 pg in the sample), depending on the analyte. Optimization of declustering potential, collision energy and CXP, was carried out for each metabolite directly injecting 1 to 5 ng of synthetic standard using the same eluent used for the analysis.

### Neutrophil elastase evaluation

Sputum supernatants were used also for NE evaluation by means of ProteaseTag® Active Neutrophil Elastase Immunoassay (Proaxis, Belfast, UK) as per manufacturer’s instructions [13].

### Real-time PCR bacterial identification

Bacterial DNA was quantified using real-time polymerase chain reaction (PCR) assay for *Pseudomonas aeruginosa* (*Psa*; gyrB gene), *Staphylococcus aureus* (*Sa*; nuc gene), *Streptococcus pneumoniae* (*Spn*; lytA gene) and *Haemophilus influenzae* (*Hi*; fucK gene) using AB7900HT Fast Real-Time PCR System (Applied Biosystems) with primers and probes previously published [14]. Quantitative PCR was performed in 20-µL reaction mixture containing 2 × QuantiFast Multiplex PCR Master Mix (Qiagen), primers and probes and 2 µL of DNA extracted from sputum samples. Moreover a real-time PCR targeting human RNaseP gene was used to detect PCR inhibition or extraction failure. All amplification were performed with parameters as follow: 95 °C for 5 min, followed by 45 cycles of 95 °C for 45 s and 60 °C for 1 min. To quantify bacterial DNA, standard curves were prepared using genomic DNA from *S. pneumoniae* (ATCC® 700669DQTM), *P. aeruginosa* (ATCC® 47085DQTM), *S. aureus* (ATCC® 29213DQTM) and *H. influenzae* (ATCC® 51907DQTM). Standard DNA were analyzed with Qubit and Quant-iT dsDNA Assay Kit High Sensitivity (Thermo Fisher Scientific) followed by dilution at 1 ng/µL. Then, standard curves were prepared by tenfold dilution from 1 ng/µL to 1*10^–7^ ng/µL, samples and curves were tested in triplicate and samples was assumed to be positive if Ct is < 38. Target genes (gyrB, nuc, lytA and fucK) were in a single copy in the genome, copies measured was assumed to be equivalent to the bacterial load [15]. The number of genome copies were calculated based on 7.22 femtograms, 3.01 femtograms, 2.28 femtograms and 1.95 femtograms of DNA per *Psa*, *Sa*, *Spn* and *Hi* genomes respectively. Quantification of bacteria in the samples were based on standard curve generate by plotting the Ct values against known genome copies. Conversion of genome copies/reaction to genome copies/mL was based on a 2µL input per reaction derived from 100µL of eluate extracted from 200µL of treated specimen. *Sa* positive samples were also analyzed by means of RIDA GENE MRSA test (r-biopharm), a multiplex real-time PCR for the identification of methicillin-resistant *Staphylococcus aureus* (MRSA) and methicillin-susceptible *Staphylococcus aureus* (MSSA).

### Clinical variables

Demographics, comorbidities, disease severity, respiratory symptoms, sputum evaluation, radiological assessment, quality of life and biological characteristics were recorded. The severity of bronchiectasis was evaluated according to both Bronchiectasis Severity Index (BSI) and the cumulative score based on five parameters: forced expiratory volume in 1 s [FEV1], age, chronic colonization, extension, and dyspnea (FACED score).

### Statistical analysis

Qualitative variables were summarized with absolute and relative (percentage) frequencies, while quantitative variables were shown with medians [interquartile ranges, (IQR)] (Table 1). Kruskal–Wallis Test, Mann–Whitney and Spearman’s correlations were used for analysis performed with SPSS statistical software, version 26.

**Table 1:** Clinical characteristics of the study population

Variables	Total cohort	Group aNE < 20 µg mL^−1^	Group aNE ⩾20 µg mL^−1^
	n = 40	n = 15	n = 25
*Demographics*			
Median (IQR) age, years	61 (53–69.5)	65 (53–74)	60 (53–67)
Females, n (%)	28 (70)	14 (93)	14 (56)
Median (IQR) BMI, kg/m^2^	23.6 (20.1–25.9)	24 (19.6–26.5)	23.5 (21–25.4)
Median (IQR) Weight, kg	61 (52–73)	61 (51–73)	62 (53–73)
Underweight (BMI < 18.5 kg/m^2^), n (%)	4 (10)	2 (13.3)	2 (8)
Former or current smoker, n (%)	10 (25)	6 (40)	4 (16)
*Disease severity*			
Median (IQR) BSI score	8 (5–12)	9 (6–11)	8 (5–12)
Median (IQR) FACED score	3 (2–4)	2 (1.5–4)	3 (2–3)
*Radiology*			
Median (IQR) Reiff score	4 (3–6)	3 (3–6)	4 (3–6)
Mean (SD) No. of lobes	3.68 (1.4)	3.66 (1.6)	3.68 (1.3)
4 + lobes involvement, n (%)	20 (50)	7 (46.6)	13 (52)
*Clinical status and diagnostic results*			
Median (IQR) total exacerbation previous year	2 (1.5–4)	3 (2–4)	2 (1–4)
≥ 2 exacerbations previous year, n (%)	30 (75)	12 (80)	18 (72)
≥ 3 exacerbations previous year, n (%)	19 (45.7)	10 (66.6)	9 (36)
≥ 1 hospitalization previous year, n (%)	7 (17.5)	2 (13.3)	5 (20)
Emphysema, n (%)	4 (10)	1 (6.66)	3 (12)
*Quality of life*			
Median (IQR) QoL-B questionnaire—Physical	53.3 (38.3–66.7)	50 (35–73.3)	56.6 (40–66.7)
Median (IQR) QoL-B questionnaire—Role	56.6 (45–73.3)	53.3 (46.7–66.7)	66.6 (41.6–73.3)
Median (IQR) QoL-B questionnaire—Vitality	44.4 (33.3–55.6)	44.4 (33.3–55.6)	55.6 (25–55.6)
Median (IQR) QoL-B questionnaire—Emotion	75 (50–91.7)	70.8 (52–83.3)	79.1 (52–91.7)
Median (IQR) QoL-B questionnaire—Social	52.8 (33.3–75)	55.6 (43.7–72.9)	45.8 (33.3–72.9)
Median (IQR) QoL-B questionnaire – Treatment Burden	55.6 (44.4–66.7)	55.6 (44.4–66.7)	55.6 (44.4–66.7)
Median (IQR) QoL-B questionnaire—Health	33.3 (20.8–52)	33.3 (27–54.8)	33.3 (16.7–48)
Median (IQR) QoL-B questionnaire—Respiration	68.5 (48.1–77.8)	68.5 (50.6–77.8)	70.4 (50–74.1)
*Relevant comorbidities*			
Median (IQR) BACI	0 (0–3)	3 (0–3)	0 (0–3)
COPD, n (%)	4 (10)	2 (13.3)	2 (8)
MRGE, n (%)	14 (35)	7 (46.6)	7 (28)
Asthma, n(%)	7 (17.5)	4 (26.6)	3 (12)
*Pulmonary function*			
Mean (SD) FEV1, %	71.3 (23.1)	71.8 (18)	68.2 (29.4)
FEV1 ≤ 35, n (%)	4 (10)	1 (6.6)	3 (12)
FEV1 ≤ 50, n (%)	7 (17.5)	1 (6.6)	6 (24)
*Chronic therapy*			
PPI, n (%)	16 (40)	8 (53.3)	8 (32)
LABA, n (%)	31 (77.5)	12 (80)	19 (76)
LAMA, n (%)	22 (55)	10 (66.6)	12 (48)
ICS, n (%)	22 (55)	7 (46.6)	15 (60)
*Sputum biomarkers*			
Median (IQR) Active neutrophil elastase, µg/ml	24.7 (13–38.5)	7 (2.1–15.2)	35.3 (28–48)
Median (IQR) LTB4, ng/mL	1.4 (0.6–4)	0.9 (0.3–1.7)	2 (0.8–4.6)
Median (IQR) PGE2, ng/mL	3.6 (1.4–5.5)	1.5 (0.9–3.8)	4.1 (2.6–5.7)
Median (IQR) 15-HETE, ng/mL	16.6 (8.6–26.6)	18.4 (6.8–25.4)	14.9 (8.9–26.3)
Median (IQR) 14-OH DHA, ng/mL	1.6 (0.91–2.6)	2.1 (1.1–5.4)	1.4 (0.8–2.1)
Median (IQR) 17-OH DHA, ng/mL	6.8 (4.1–11.7)	7.3 (4.8–21.5)	5.9 (2.7–10.2)
Median (IQR) LTE, ng/mL	0.17 (0.0–0.4)	0.17 (0.0–0.4)	0.18 (0.0–0.4)
*Real Time PCR Bacterial Identification*			
Positive to Psa, n (%)	21 (52.5)	5 (33.3)	16 (64)
Median (IQR) Psa genome copies*mL^−1^	2,583,621.9 (0–106,128,808.9)	0 (0–10,530,297.8)	42,243,767 (0.0–145,083,102.5)
Positive to Hi, n (%)	0 (0)	0 (0)	0 (0)
Median (IQR) Hi genome copies*mL^−1^	0 (0–0)	0 (0–0)	0 (0–0)
Positive to Sa, n (%)	16 (40)	6 (40)	10 (40)
MRSA, n (%)	4 (10)	1 (6.6)	3 (12)
Median (IQR) Sa genome copies*mL^−1^	0 (0–5031.1)	0 (0.0–5602.1)	0 (0.0–4053.1)
Positive to Spn, n (%)	11 (27.5)	4 (26.6)	7 (28)
Median (IQR) Spn genome copies*mL^−1^	0 (0–276.9)	0 (0.0–268.2)	0 (0.0–245.4)

*BMI*  body mass index, *BSI*  bronchiectasis severity index, *FACED*  F: Forced expiratory volume in 1 s [FEV1], *A* age, *C* chronic colonization by *Pseudomonas aeruginosa*, *E* radiological extension [number of pulmonary lobes affected], *D* dyspnea, *BACI*  bronchiectasis aetiology comorbidity index, *COPD*  chronic obstructive pulmonary disease, *MRGE*  gastro-esophageal reflux disease, *FEV1*  forced expiratory volume in the 1st second, *PPI*  proton pump inhibitors, *LABA*  long-acting β2-agonists, *LAMA*  long-acting muscarinic antagonists, *ICS*  inhaled corticosteroids

## Results

Forty adults [28, (70%) female, age median (IQR): 61 (53–69.5)] were enrolled. Characteristics of the cohort, including active neutrophil elastase (aNE), bacteria identification and related genome copies, and AA and DHA metabolites in sputum samples are reported in Table 1. Mass spectrometry analysis showed the presence of pro-inflammatory mediators such as LTB_4_ [median (IQR): 1.4 ng/mL (0.6–4)], PGE_2_ [median (IQR): 3.6 ng/mL (1.4–5.5)], 15-HETE [median (IQR): 16.6 ng/mL (8.6–26.6)] and LTE_4_ [median (IQR): 0.17 ng/mL (0.0–0.4)] as well as pro-resolving mediators precursors 14OH-DHA [median (IQR): 1.6 ng/mL (0.91–2.6)] and 17OH-DHA [median (IQR): 6.8 ng/mL (4.1–11.7)]. Active neutrophil elastase had a median (IQR) concentration of 24.7 µg/mL (13–38.5). Real-time PCR allowed to identify bacteria and related genome copies (% of detection; median copies with IQR) such as *Psa* [52.5%; 2,583,621.9 (0–106,128,808.9)], *Hi* [0%; 0 (0–0)], *Sa* [40%, 0 (0–5031.1); 10% MRSA] and *Spn* [27.5%, 0 (0–276.9)].

### Fatty acid pro-resolving/pro-inflammatory mediators versus clinical and laboratory data

**Fig. 1:**
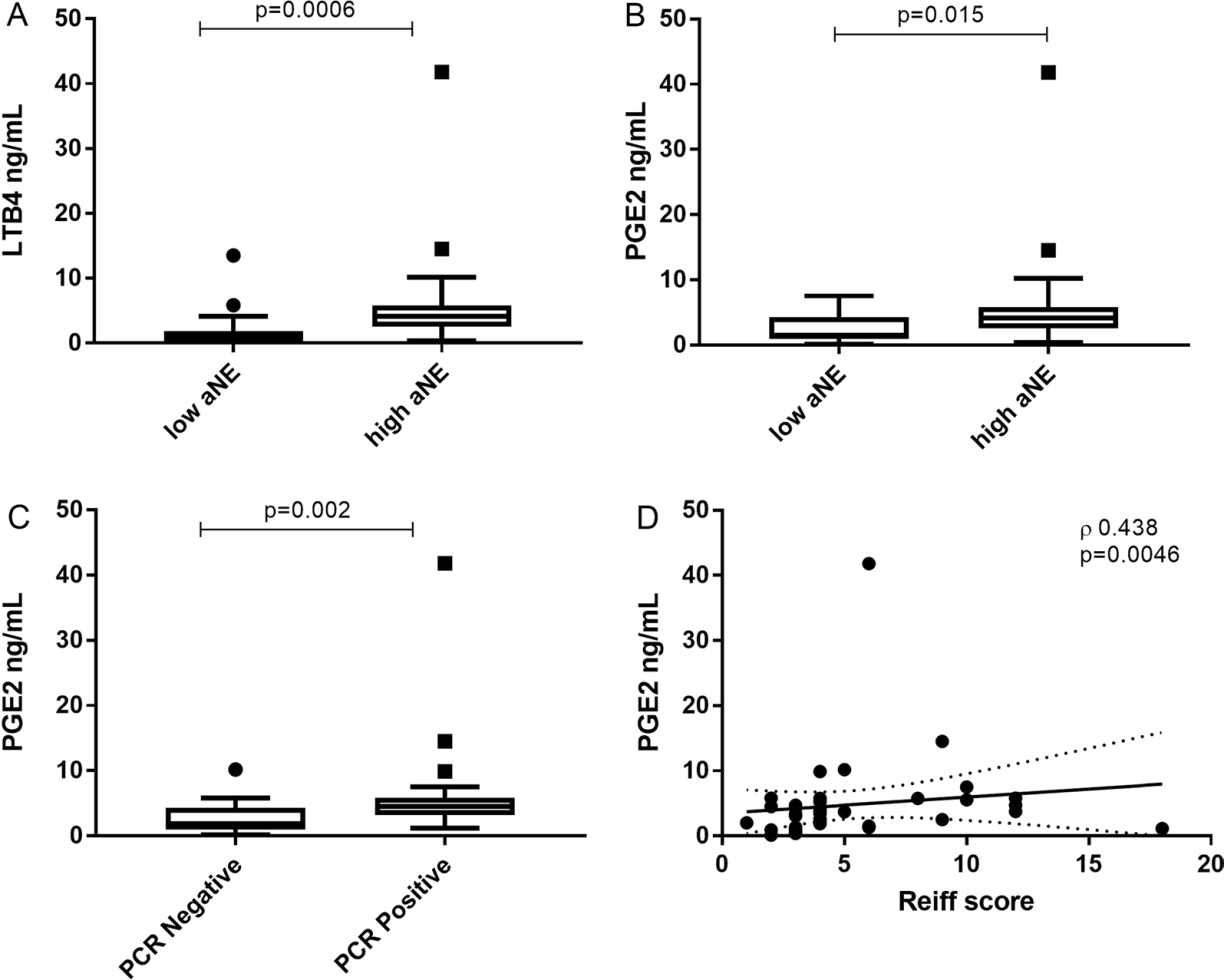
LTB_4_ (Panel **A**) and PGE_2_ (Panel **B**) in sputum samples with high or low levels of active neutrophil elastase (aNE). Panel **C**: PGE_2_ in sputum samples with or without *Pseudomonas aeruginosa* infection. Panel **D**: Correlation between PGE_2_ in sputum samples and radiological severity

Patients were divided into two groups based on the concentration of aNE as defined by Chalmers and collegues [16] of being most associated with worse outcomes in bronchiectasis: aNE ⩾20 µg·mL^−1^ (high aNE) versus aNE < 20 µg mL^−1^ (low aNE). Analysis of pro-inflammatory biomarkers, in particular LTB_4_ [low aNE: 0.92 ng/mL (0.30–1.71) vs. high aNE: 2.07 ng/mL (0.84–4.59); p = 0.0006, Fig. 1A] and PGE_2_ [low aNE: 1.51 ng/mL (0.96–3.87) vs. high aNE: 4.14 ng/mL (2.60–5.75); p = 0.015, Fig. 1B] revealed significantly higher concentrations in the high aNE group. Moreover PGE_2_ was also statistically higher in samples positive for *Psa* [negative PCR: 1.95 ng/mL (0.93–4.26) vs. positive PCR: 4.51 ng/mL (3.36–5.79); p = 0.002, Fig. 1C], and showed a significantly correlated with radiological severity as measured by Reiff score (ρ:0.438, p = 0.0046, Fig. 1D). Finally, 15-HETE correlated with the number of exacerbations in the previous year (ρ:0.343, p = 0.029), and taken together all these evidence suggested a close link between AA-derived lipid mediators and clinical inflammatory markers.

**Fig. 2:**
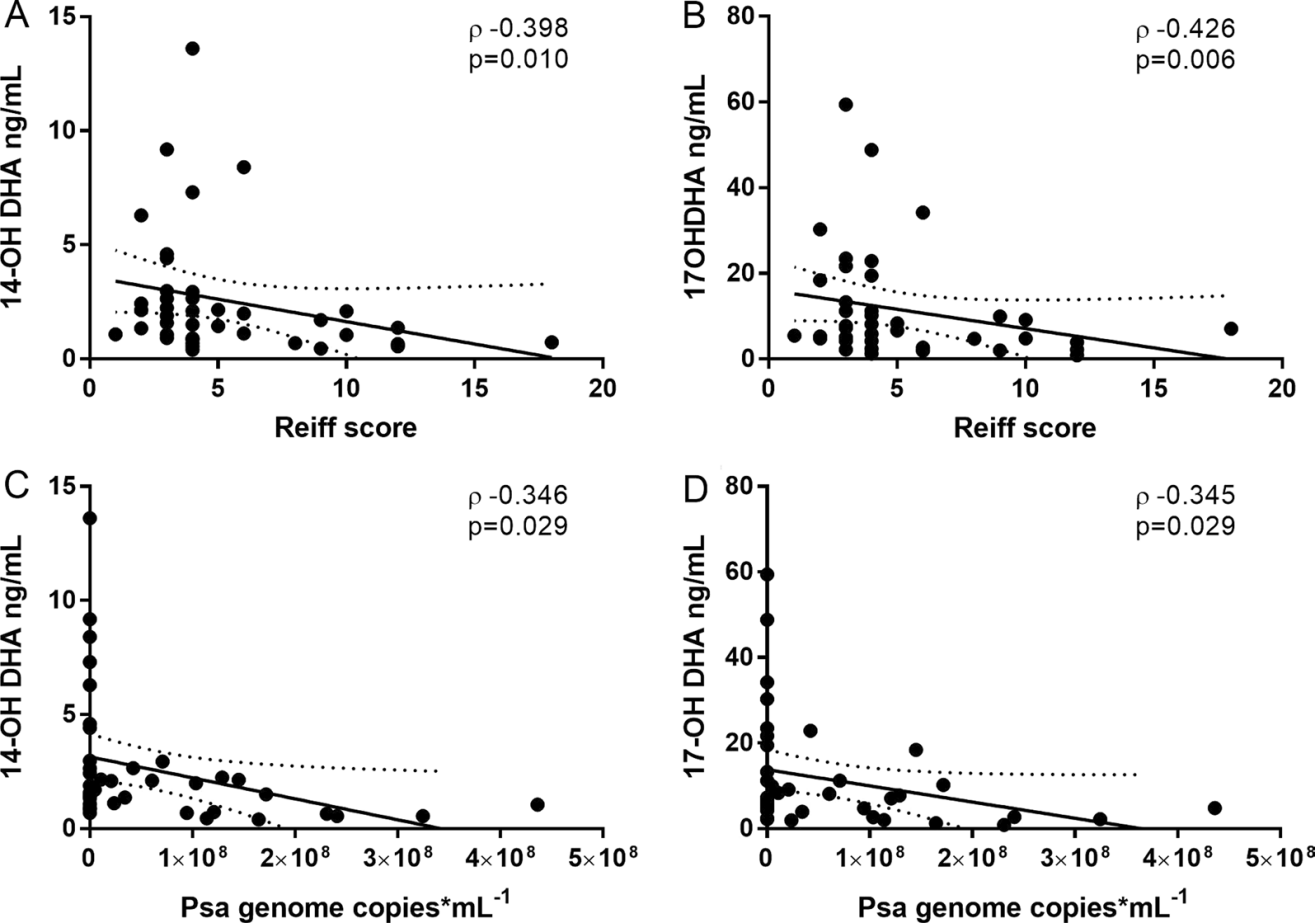
Inverse correlation between 14OH-DHA or 17OH-DHA, and radiological severity as assessed by Reiff score (Panels **A** and **B**, respectively), or PSA genome copies (Panels **C** and **D**, respectively)

On the contrary, the precursor of SPMs 14OH-DHA and 17OH-DHA showed statistically significant inverse correlations with Reiff score [Reiff score 14OH-DHA (ρ: − 0.398, p = 0.010), Fig. 2A; Reiff score 17OH-DHA (ρ: − 0.426, p = 0.006), Fig. 2B] and with detected *Psa* genome copies [*Psa* 14OH-DHA (ρ: − 0.346, p = 0.029), Fig. 2C; *Psa* 17OH-DHA (ρ: − 0.345, p = 0.029), Fig. 2D], and 14OH-DHA concentrations also showed inverse correlation with daily sputum volume production (ρ: − 0.445, p = 0.004).

## Discussion

In agreement with previously published data [16–18], the findings of the present work provide evidence that the presence of *Psa* in sputum of bronchiectasis patients may drive airway neutrophilic inflammation possibly through the formation of the potent neutrophil chemotactic factor LTB_4_ [19], leading to a significant influx and activation of neutrophils with theassociated release of active neutrophil elastase. PGE_2_ may also contribute to the chemotactic effect of LTB_4_ by inducing vasodilation, and its correlation with the Reiff score also suggests a role in radiological severity. These results are well in agreement with a recent report assessing eicosanoids in bronchoalveolar lavage fluids of patients with mild to severe bronchiectasis [20], and are consistent with the known role of AA-derived lipid mediators in the inflammatory response [21]. Results from the analysis of precursors of pro-resolving mediators such as 14OH-DHA and 17OH-DHA showed significant inverse correlations of their concentrations in sputum with clinical parameters such as the Reiff score, with detected *Psa* genome copies, and with daily sputum volume production. Bedi and colleagues [20] also reported the measurement of eicosanoids in plasma, suggesting that in bronchiectasis patients lower concentrations of LXA_4_, a trihydroxyderivative of AA endowed with anti-inflammatory activity [22], may contribute to the severity of the disease. This evidence is also in line with our findings, but it must be noted that plasma determination of biologically active AA-derived metabolites may not be appropriate to assess the formation of these metabolites in vivo [23]. Eicosanoids are local mediators produced on-demand, that do not circulate, as hormones do, in order to reach their targets, but act directly in the local environment, typically in the immediate surroundings of the cells synthesizing it. Furthermore, they are very often locally metabolized either by spontaneous chemical degradation, as for Prostaglandin I_2_ and Thromboxane A_2_ [24], or by enzymatic activities, such as for the formation of 20-hydroxy and 20-carboxy derivative of LTB_4_ by neutrophil themselves [25] or macrophages. When entering the circulation PGE_2_, for example, is completely metabolized by a single pulmonary transit [26], and it is generally accepted that when local sampling is not possible, systemic formation of eicosanoids should be assessed measuring the concentration of their urinary metabolites [27–29].

While we believe this study is of interest in defining critical factors for the evolution of the pathology in different subjects, we are also aware that it comes with some limitations: (1) the number of subjects is reduced and sample size cannot be calculated due to absence of other studies on fatty acid mediators using sputum from bronchiectasis patients; (2) data on diet was not recorded during observation and this might be important because omega-3 and omega-6 fatty acids intake could change the availability of AA and DHA as substrates of pro-inflammatory or pro-resolution mediators; (3) sputum has been selected as matrix of interest because of the local nature of the mediators studied but significant variability may be present; (4) the study is monocentric and data may not be generalized.

## Conclusion

The observation that SPM precursor molecules could be detected locally, in sputum samples from bronchiectasis patients, and inversely correlate to severity, as in the case of the Reiff score, suggest that DHA-derived metabolites may play a role helping the resolution of the inflammatory reaction and limiting pulmonary damage. Evidence that SPMs can enhance clearance of microorganisms and stimulate tissue repair, as well as modulate viral and bacterial infections by increasing phagocytosis and the ability to kill bacteria [30, 31] support the observed inverse correlation of SPM precursor molecules with detected *Psa* genome copies.

This study can be taken as a pilot for additional longitudinal studies in which subjects could be included based on (i.e.) stable versus exacerbation state; additionally the effect of DHA supplementation could also be evaluated with respect to both the formation of DHA-derived oxygenated metabolites and clinical parameters including Reiff score and *Psa* colonization. This approach could be useful to better understand the specific weight of pro-inflammatory vs. pro-resolution mediators, and to assess the ability of DHA supplementation to affect the duration of exacerbation and total number of exacerbations during year.

## Data Availability

The datasets used and/or analysed during the current study are available from the corresponding author on reasonable request.

## Abbreviations

*Psa*: *Pseudomonas aeruginosa*
ROS: Reactive oxygen species
Lms: Lipid mediators
NE: Neutrophil elastase
CF: Cystic fibrosis
DHA: Docosahexaenoic acid
LA: Linoleic acid
AA: Arachidonic acid
SPMs: Specialized pro-resolving mediators
PUFAs: Polyunsaturated fatty acids
LTB_4_: Leukotriene B_4_
PGE_2_: Prostaglandin E_2_
5-HETE: 5-Hydroxyeicosatetraenoic acid
LXA_4_: Lipoxin A_4_
17OH-DHA: 17-Hydroxy-docosahexaenoic acid
PD1: Protectin D1
15-HETE: 15-Hydroxyeicosatetraenoic acid
LTE_4_: Leukotriene E_4_
RvD1: Resolvin D1
14OH-DHA: 14-Hydroxy-docosahexaenoic acid
MaR1: Maresin 1
DNA: Deoxyribonucleic acid
PCR: Polymerase chain reaction
*Sa*: *Staphylococcus aureus*
*Spn*: *Streptococcus pneumoniae*
*Hi*: *Haemophilus influenzae*
ct: Cycle threshold
aNE: Active neutrophil elastase
gyrB: Subunit B DNA gyrase
nuc: Nuclease
lytA: Autolysin
fucK: Fuculokinase
BMI: Body mass index
BSI: Bronchiectasis severity index
BACI: Bronchiectasis aetiology comorbidity index
COPD: Chronic obstructive pulmonary disease
MRGE: Gastro-esophageal reflux disease
FEV1: Forced expiratory volume in the 1st second
PPI: Proton pump inhibitors
LABA: Long-acting β2-agonists
LAMA: Long-acting muscarinic antagonists
ICS: Inhaled corticosteroids
